# Individual Species-Area Relationship of Woody Plant Communities in a Heterogeneous Subtropical Monsoon Rainforest

**DOI:** 10.1371/journal.pone.0124539

**Published:** 2015-04-17

**Authors:** Cheng-Han Tsai, Yi-Ching Lin, Thorsten Wiegand, Takefumi Nakazawa, Sheng-Hsin Su, Chih-Hao Hsieh, Tzung-Su Ding

**Affiliations:** 1 School of Forestry and Resource Conservation, National Taiwan University, Taipei, Taiwan; 2 Institute of Oceanography, National Taiwan University, Taipei, Taiwan; 3 Department of Life Science, Tunghai University, Taichung, Taiwan; 4 Department of Ecological Modelling, UFZ Helmholtz Centre for Environmental Research, Leipzig, Germany; 5 Department of Life Sciences, National Cheng Kung University, Tainan, Taiwan; 6 Taiwan Forestry Research Institute, Taipei, Taiwan; 7 Institute of Ecology and Evolutionary Biology, National Taiwan University, Taipei, Taiwan; Beijing Normal University, CHINA

## Abstract

The spatial structure of species richness is often characterized by the species-area relationship (SAR). However, the SAR approach rarely considers the spatial variability of individual plants that arises from species interactions and species’ habitat associations. Here, we explored how the interactions of individual plants of target species influence SAR patterns at a range of neighborhood distances. We analyzed the data of 113,988 woody plants of 110 species from the Fushan Forest Dynamics Plot (25 ha), northern Taiwan, which is a subtropical rainforest heavily influenced by typhoons. We classified 34 dominant species into 3 species types (i.e., accumulator, repeller, or no effect) by testing how the individual species-area relationship (i.e., statistics describing how neighborhood species richness changes around individuals) of target species departs (i.e., positively, negatively, or with no obvious trend) from a null model that accounts for habitat association. Deviation from the null model suggests that the net effect of species’ interactions increases (accumulate) or decreases (repel) neighborhood species richness. We found that (i) accumulators were dominant at small interaction distances (<10–30 m); (ii) the detection of accumulator species was lower at large interaction distances (>30 m); (iii) repellers were rarely detected; and (iv) large-sized and abundant species tended to be accumulators. The findings suggest that positive species interactions have the potential to accumulate neighborhood species richness, particularly through size- and density-dependent mechanisms. We hypothesized that the frequently disturbed environment of this subtropical rainforest (e.g., typhoon-driven natural disturbances such as landslides, soil erosion, flooding, and windthrow) might create the spatial heterogeneity of species richness and promote positive species interactions.

## Introduction

Understanding the spatial distribution of species diversity is a fundamental goal of community ecology. Spatial patterns of species diversity are driven by biotic and abiotic factors that govern community development, such as species interactions, dispersal, and environmental disturbance [1–6]. Analyses that explicitly consider the spatial structure of species diversity facilitate the generation of hypotheses about factors that regulate the community [7–9]. A large body of studies has characterized species diversity over space using the species-area relationship (SAR) [6, 10–11]. The SAR quantifies how species richness changes with sampling area from a “plot-centered” perspective, namely, sampling area bounded by a given geometrical shape [11]. However, spatial heterogeneity of the SAR has been largely overlooked. If spatial structure (e.g., aggregation or over-dispersion) of individuals was integrated in the conventional SAR, then spatial heterogeneity might be quantified via the SAR approach [10, 12, 13]. One way to extend the limitation of the “plot-centered” SAR is to analyze the community from the viewpoint of individuals, providing a “plant’s-eye” view of the community [13]. This individual species-area relationship (hereafter ISAR; note that the abbreviation ISAR has been used in other SAR studies with different meanings; e.g., [14–15]) approach estimates the number of species within (circular) plots of a given size around individuals of target species. Accordingly, spatial heterogeneity caused by individual interactions or environmental factors may be estimated to examine community level consequences through the ISAR [13], providing an opportunity to generate hypotheses about community structure.

The ISAR also provides an individual-based perspective for investigating the spatial scale of different ecological effects. The processes that shape the spatial distribution of species operate at different spatial scales around individuals [16–19]. For example, species composition at small spatial scales might be strongly influenced by intra-specific competition or inter-specific interactions between neighborhing individuals [3, 17]. In contrast, at large spatial scales, the emergence of distinct species composition might result from species responses to environmental conditions in a patchy way [20–22]. These scale-dependent effects are key components of spatial variability that might be associated with the spatial distributions of individuals and the spatial heterogeneity of species diversity on the landscape. However, these effects have rarely been investigated using the conventional SAR approach.

The ISAR may be extended to investigate the effects of individual size and neighborhood abundance. For example, the inter- and intra-specific interactions within neighborhoods in plant communities may be mediated by differences in the size of individuals [3, 17, 23–25]. Large-sized individuals may asymmetrically out-compete small-sized individuals in a neighborhood by shading the light [23]. In parallel, small-sized individuals may be sensitive to herbivores and pathogens transmitted from large-sized individuals in the neighborhood [3, 25]. Other size-mediated processes, such as disturbance-driven size filtering of communities [26], may generate additional spatial heterogeneity. Moreover, these size-mediated species interactions may depend on the neighborhood distance to individuals or the spatial density of inter- and intra-specific individuals. For example, the Janzen—Connell hypothesis emphasizes mechanisms of distance-, density-, and size/age-dependence of community structuring in a spatial context [3, 25]. That is, large-sized individuals (adults) tend to accumulate heterospecific small-sized individuals (saplings) in their neighborhood because conspecific seedlings/saplings in the neighborhood of large-sized individuals (adults) suffer higher mortality than conspecific seedlings/saplings in the neighborhood of small-sized individuals [3]. In addition to the presence of conspecific adults, mortality of saplings might also be affected by the abundance of conspecific adults. Thus, size differences among individuals and neighborhood abundance may be correlated with species richness on the landscape. Consequently, size differences among individuals and neighborhood abundance may co-mediate the spatial heterogeneity that should be detected by ISAR.

Investigating the ISAR in a disturbance-driven community could improve our understanding about species interactions, and in particular, those that are size- and/or abundance-dependent. In this study, the ISAR approach was applied to a woody plant community in a frequently disturbed subtropical rainforest of northern Taiwan. Our dataset is appropriate for applying the ISAR approach because the location of each individual plant, in addition to species identity and individual size, has been recorded [27]. In addition, the woody plant community is subject to frequent natural disturbances primarily driven by typhoon induced flooding, landslides, soil-erosion, and wind-induced branch damage [28, 29]. These recurrent disturbances consistently contribute to spatial heterogeneity via changes to the size distribution of individuals, neighborhood abundance/density, and hydro-geochemical variables at large spatial scales [22, 30]. These disturbances may also increase sprouting and litterfall around individual plants, which, in turn, create heterogeneous environments that enhance neighborhood species richness [26, 28, 29]. Previous study showed that signals of species interactions (i.e., nonrandom neighborhood species richness) do not tend to be dominant in species-rich tropical forests [13]. In contrast, neighborhood species richness might be driven by the distinct nature of plant interactions that has arisen from disturbance-induced spatial heterogeneity [4, 5, 28].

The recurrent disturbances at our study site provide an opportunity to investigate whether disturbance-induced spatial heterogeneity promotes or weakens individual interactions in ISAR patterns, particularly with respect to interactions that are dependent on size and abundance of individuals. Specifically, we examined: (i) whether neighborhood species richness around the individuals of target species departed from the null expectation of SAR; (ii) if target species act as accumulator or repeller species, or show no effect, on neighborhood species richness; (iii) whether interactions generating accumulator and repeller species depend on interaction distances; and, (iv) if accumulator or repeller species are correlated with individual size and abundance.

## Materials and Methods

### Field data

**Fig 1:**
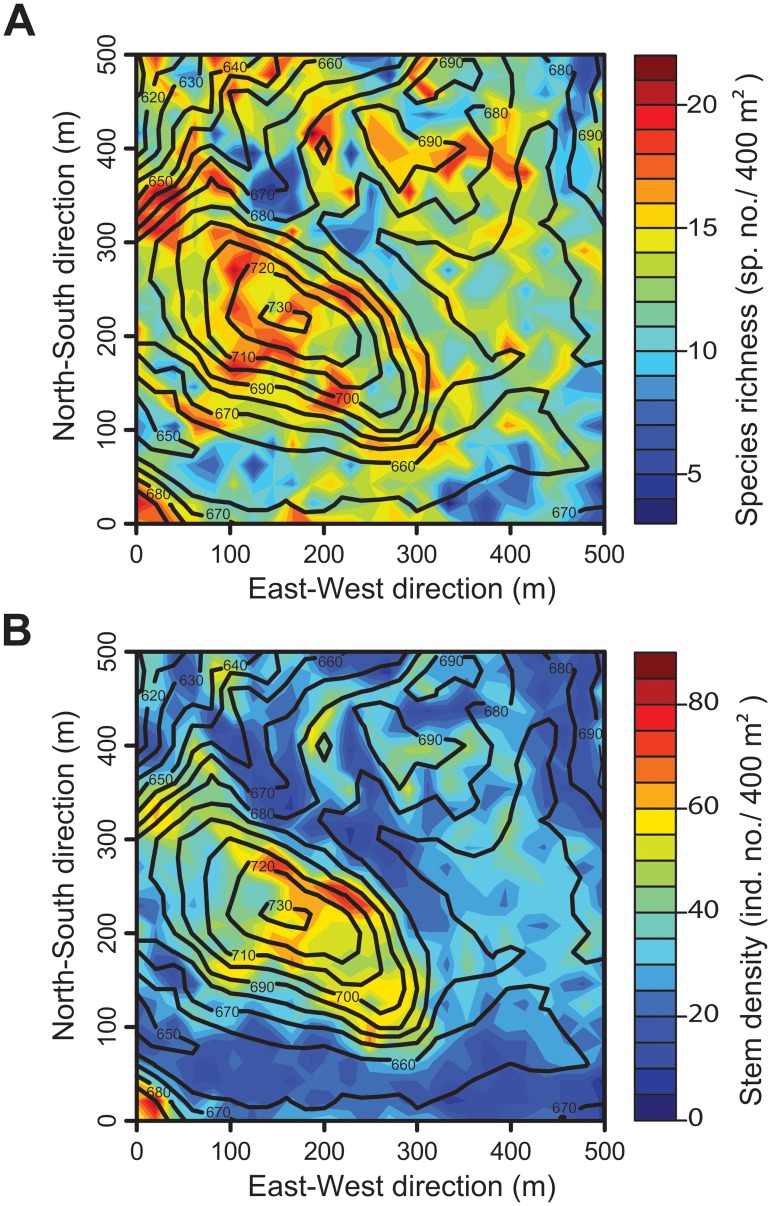
Fushan Forest Dynamic Plot. Spatial variations in (A) species richness and (B) woody plant density. Contour lines indicate the elevation (m.a.s.l.). Individuals with diameter at breast height >10 cm are shown.

Our research site was the 25 ha Fushan Forest Dynamics Plot (FFDP) in northern Taiwan (24°45’40” N, 121°33’38” E). At this site, individual plants with a diameter >1 cm at breast height (DBH) have been recorded and mapped using two-dimensional spatial coordinates since 2003. The vegetation dataset of FFDP is provided and maintained by the Taiwan Forestry Research Institute (TFRI). The dataset is open to the public and other researchers can obtain the same data by a formal application to TFRI. Readers may contact Sheng-Hsin Su (sush@tfri.gov.tw) for further information [27]. The study area has an average annual temperature of about 18°C, and average annual precipitation of over 4,200 mm delivered by frequent heavy typhoons in summer, and the northeast monsoon in winter [27, 29]. The elevation of the study site ranges from 600 m to 733 m above sea level. The species richness and density of individual woody plants are relatively high (i.e., >15 species, and >40 individuals per 400 m^2^) in high-elevation areas (Fig 1A and 1B). A previous study showed that many species at this site exhibit strong habitat associations related to wind-induced disturbances [28]. In this study, we used FFDP data that included the locations of 110 species recorded in 2003 and 2004. Individual plants were divided into 18,750 adults (above 10 cm DBH) and 95,238 saplings (1–10 cm DBH). We then extracted the spatial coordinates of adult individuals of major species (i.e., species abundance >50 individuals; note that the choice of this abundance level is for comparison of previous tropical forest studies and is based on the sensitivity analysis; see S1 Text). In our main analysis (i.e., large-large relationships), we considered adults only to examine the long-term history of forest dynamics. In addition, we used the spatial coordinates of small-sized saplings of all species to investigate the size dependent effect of interactions (i.e., large-small relationships).

### ISAR

We used the individual species-area relationship function *ISAR*_*t*_(*r*), which is defined as the mean number of species within radius *r* of individuals of a target species *t*:

$$ISAR_{t}(r)=\sum_{j\neq t}^{S}D_{tj}(r),$$

where the cumulative nearest neighbor distribution function *D*_*tj*_(*r*) is the proportion of individuals of target species *t* that have the nearest-neighbor of species *j* within distance *r*, and total number of species is denoted by *S*. The ISAR function describes how each species contributes to species richness at a given neighborhood distance (spatial scale). One important advantage of this approach is that it may be used to explore the net effects of inter-specific interactions at the community level without detailed information on pairwise relationships. Note that the ISAR function was designed to focus on species richness patterns, but it may be modified to evaluate other dimensions of diversity (e.g., phylogenetic or functional diversity) [31]. Here, we assessed the *ISAR*_*t*_(*r*) of 34 target species by varying the neighborhood radius *r* from 1 to 50 m at intervals of 1 m. We estimated the *D*_*tj*_(*r*) without edge correction because the bivariate patterns of species *t* and *j* are not necessarily homogeneous, which causes problems with current edge correction methods [32]. In addition, the small bias caused by focal individuals that are located near borders is averaged out, because all of the empirical ISAR functions and the null model simulations are subject to the same bias. We compared the observed ISAR functions of the 34 target species to 2 null models (see below). That is, first, we tested whether a target species was located in areas of lower or higher local species richness compared to that expected by the average SAR. Second, we tested whether the local species richness around a target species was driven by individual interactions after approximately accounting for species’ habitat associations and, if so, at which neighborhood scales individual interactions occurred [13]. Monte Carlo tests for significance under null models required intensive computing capacity. Therefore, significance was tested by generating 199 Monte Carlo simulations for each target species and by determining whether the empirical *ISAR*_*t*_(*r*) was above or below the 95% quantile value of the simulated *ISAR*_*t*_(*r*) (example for a representative species; see S1 Fig). Sampling effect, due to varying abundance of a target species, may influence the power of the Monte Carlo test for detecting significant departures from the null models. Thus, we performed sensitivity analysis to determine whether variation in the individual abundance of each target species would affect the power of ISAR for detecting significant departures from the null models (see S1 Text and S2 Fig for details of the method used).

### Homogeneous Poisson null model

We used a homogeneous Poisson process to compare the observed neighborhood species richness around individuals of a target species with that of neighborhoods at random locations across the entire study area. Individuals of a target species were randomly redistributed over the entire landscape of the study site (i.e., the point pattern was analogous to conventional SAR [10]), while the locations of other species remained fixed. By comparing the observed and simulated ISAR, we determined how the ISAR of each target species deviated from the conventional SAR. Because the random samples were taken from all habitats occurring in the plot, we expect that deviations of SAR that are out of the range of individual interactions would be mostly driven by habitat filtering. The homogeneous null model was used as a baseline against which the heterogeneous Poisson null model (which approximately accounts for species’ habitat associations) was compared. Through comparison of departures from the two null models, we evaluated the “pure” effect of individual interactions that generate the spatial heterogeneity for SAR patterns [13, 32–34].

### Heterogeneous Poisson null model

We used a heterogeneous Poisson null model to evaluate the effect of species interactions on ISAR. This approach is based on the assumption that species interactions and species’ habitat associations do not occur at the same spatial scale, known as “separation of scales” ([19]; see S2 Text and S4 Fig for details of the assumption and sensitivity tests). We assumed that the spatial distributions of individuals reflected species’ habitat associations at large scales and individual interactions at small scales [13, 19]. Based on this assumption, the heterogeneous null model redistributes individuals locally within a given neighborhood radius to similar habitats, and therefore maintains the observed large-scale species habitat association for each target species (see comparison to other randomization methods; [35]). We used a maximum redistribution radius of 50 m to compare our findings with those of previous studies [13, 36], and to consider large-scale disturbance at our study site (e.g., disturbance-induced canopy gaps, and hydrological or topographic modifications that often occur at >50 m scales; [28]). We implemented the heterogeneous Poisson null model by using a non-parametric kernel estimation of the spatially varying intensity functions of the target species (see S2 Text for methodological details). Specifically, the non-parametric kernel estimation was constructed using a distance-weighted moving window around individual locations. In addition, we performed sensitivity analysis to ensure that variation in the values of the maximum radius of kernel estimation did not affect ISAR patterns (S5 Fig). Finally, species with significantly positive (or negative) deviation of empirical ISAR from that expected in the heterogeneous null model were classified as accumulators (or repellers) at a given spatial scale. Species with ISAR that did not deviate significantly from the model were classified as “no effect” species.

### Goodness-of-fit tests for null models

We conducted goodness-of-fit tests to reduce Type I error inflation due to multiple testing [37]. We first evaluated the accumulated deviations *u*_i_ with *i* = 0 for the observed ISAR function and *i* = 1,…, 199 for null model simulations. The index *u*_i_ represents the accumulated deviation of the observed ensemble statistics from the expected ensemble statistics under the null model, which was summed over an appropriate distance interval (*r*_min_, *r*_max_):

$$u_{i}=\sum_{r=r_{\min}}^{r_{\max}}{\left({\hat{H}}_{i}(r)-\bar{H}(r)\right)}^{2}$$

where ${\hat{H}}_{i}(r)$ is an observed (*i* = 0) or simulated (*i* = 1–199) *ISAR*_*t*_(*r*) function, and $\bar{H}\left(r\right)$ is the averaged ensemble statistics, excluding the *i*th function (i.e.,$\frac{1}{199}\sum_{j\neq i}{\hat{H}}_{j}(r)$). Note that ${\bar{H}}_{0}(r)$ yields the averaged ensemble statistics of all simulated patterns and, thus, provides an unbiased estimate of *H*(*r*) under the null models [35]. The rank of the statistic *u*_0_ of the observed ISAR among all *u*_*i*_ was used for the goodness-of-fit test, so that a significant departure from the null model occurred for *α* = 0.05 (i.e., the rank of *u*_0_ was larger than 190). We assessed *u*_i_ at 5 distance intervals: 1–10 m, 11–20 m, 21–30 m, 31–40 m, and 41–50 m.

### Plant size and abundance effect on ISAR estimation

We evaluated: (i) whether the ISAR results vary with size differences between target and neighborhood plants, and (ii) if ISAR results of a target species are associated with the abundance rank. We used the heterogeneous Poisson process (which accounts for species habitat associations at >50 m scale) as the null model to determine whether the effects of individual interactions are influenced by size differences. Ideally, species-specific size frequency distributions would be determined for all species and related to their spatial coordinates. However, this is empirically and computationally challenging. Therefore, we evaluated large-small relationships between adults (>10 cm DBH) and saplings (1–10 cm DBH); that is, we considered adults as targets and saplings as neighborhood species (using the same methodology as for the large-large relationships). Then, we compared the large-large relationships with the large-small relationships under the heterogeneous null model to evaluate how size differences among adjacent individuals affect ISAR patterns. Note that we assumed the separation of scales with species’ habitat associations at >50 m and individual interactions at <50 m when creating heterogeneous null models. Thus, the potential effects of small-scale habitat associations were not considered in the comparison between large-large and large-small relationships. Instead, differences between large-large and large-small relationships were assumed to result purely from the effects of individual interactions. We used a diagram in which the ISAR of target species was categorized according to species’ abundance rank to evaluate the effect of individual abundance on ISAR types under the null models.

## Results

**Fig 2:**
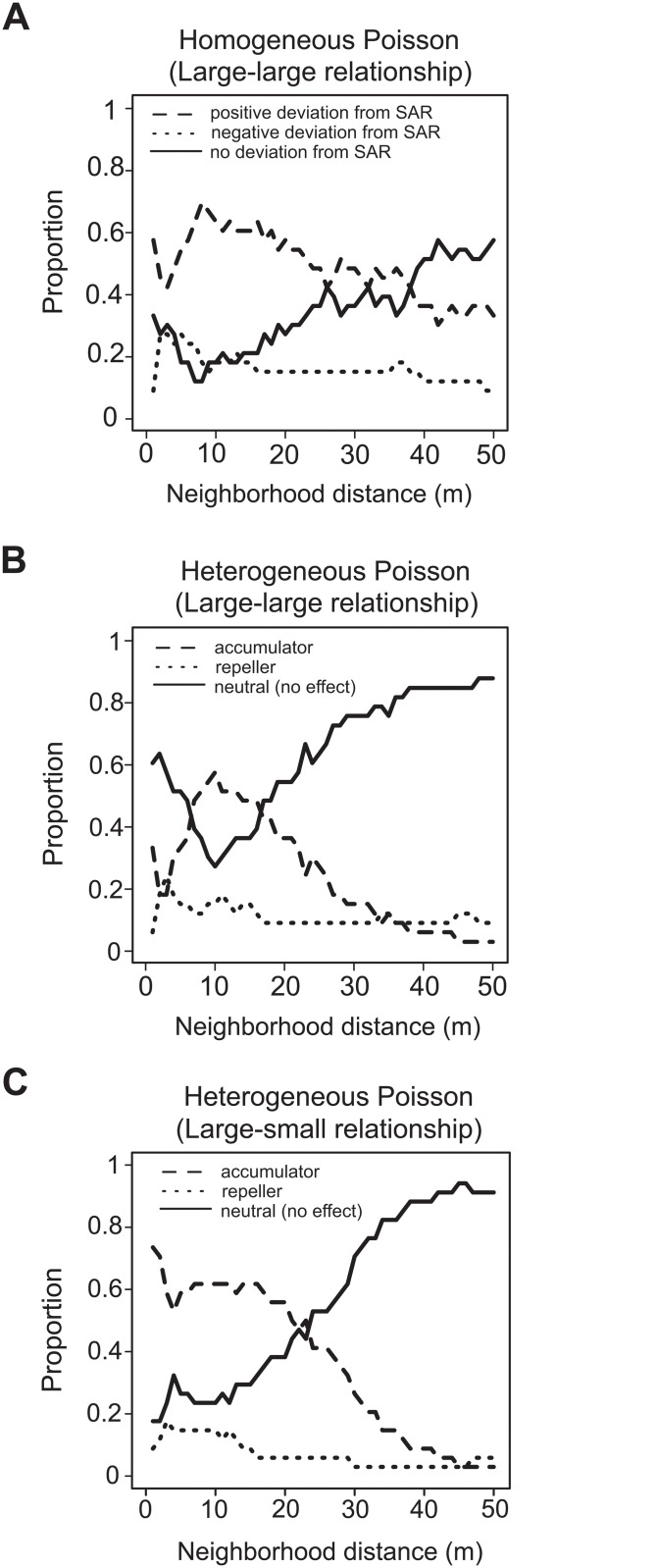
Proportion of species types along neighborhood distances. (A) Homogeneous and (B) heterogeneous Poisson null models for “large-large relationships” and (C) heterogeneous Poisson null model for “large-small relationships.” The broken, dotted, and solid lines indicate the positive, negative, and no deviation from conventional SAR in (A) and the accumulator, repeller, and no effect types in (B, C), respectively.

The 34 target species were classified into 3 types: positive, negative, and no deviation from SAR (homogeneous Poisson null model; Fig 2A) and accumulator, repeller, and no effect (heterogeneous Poisson null models; Fig 2B) (see S1 Table for each species classification). Positive departures from the null models were the most dominant type at neighborhood distances <10–30 m, whereas most target species showed no deviation from the null models at neighborhood distances >30 m. We found only a few target species that were repeller species. The ISAR goodness-of-fit tests for the large-large relationships produced consistent results. Specifically, under the homogeneous Poisson model, a high proportion of species with positive departures from the SAR were detected at relatively small neighborhood distances (62%, 62%, and 47% for 1–10 m, 11–20 m, and 21–30 m, respectively; Table 1). Under the heterogeneous Poisson model, the proportions of accumulator and “no effect” species were comparable at neighborhood distances <20 m, whereas the no effect type was dominant at large neighborhood distances (68%, 85%, and 88% for 21–30 m, 31–40 m, 41–50 m, respectively; Table 1). Repeller species were rare at all neighborhood distances (≤ 18%; Table 1).

**Table 1:** Summary of goodness-of-fit tests on individual species-area relationships for various neighborhood distance classes.

	Neighborhood distance classes (m)				
	1–10	11–20	21–30	31–40	41–50
**Homogeneous Poisson**					
Positive deviation from SAR	21 (0.62)	21 (0.62)	16 (0.47)	12 (0.35)	10 (0.30)
Negative deviation from SAR	8 (0.23)	6 (0.18)	5 (0.15)	6 (0.18)	4 (0.12)
No deviation from SAR	5 (0.15)	7 (0.20)	13 (0.38)	16 (0.47)	20 (0.58)
**Heterogeneous Poisson**					
Accumulator	16 (0.47)	15 (0.44)	8 (0.23)	2 (0.06)	1 (0.03)
Repeller	6 (0.18)	4 (0.12)	3 (0.09)	3 (0.09)	3 (0.09)
No effect	12 (0.35)	15 (0.44)	23 (0.68)	29 (0.85)	30 (0.88)

Species richness (with proportion of total richness) of the 3 types are shown: positive, negative, and no deviation from conventional SAR under the homogeneous Poisson null model, and accumulator, repeller, and no effect under the heterogeneous Poisson null model.

There was a quantitative difference between the proportion of species with positive departures from SAR and accumulator species (Fig 2A and 2B). Compared with positive departures under the heterogeneous Poisson model, a greater number of positive departures were detected under the homogeneous Poisson null model at neighborhood distances >30 m (Fig 2A and 2B). These quantitative differences may be attributed to large-scale (>50 m) spatial heterogeneity (i.e., species habitat associations) that was accounted for in the heterogeneous null model. Under the assumption of separation of scales, we found that accumulators were driven by “pure” biotic individual interactions at neighborhood distances <10–30 m and by species’ habitat associations at neighborhood distances >30 m.

**Fig 3:**
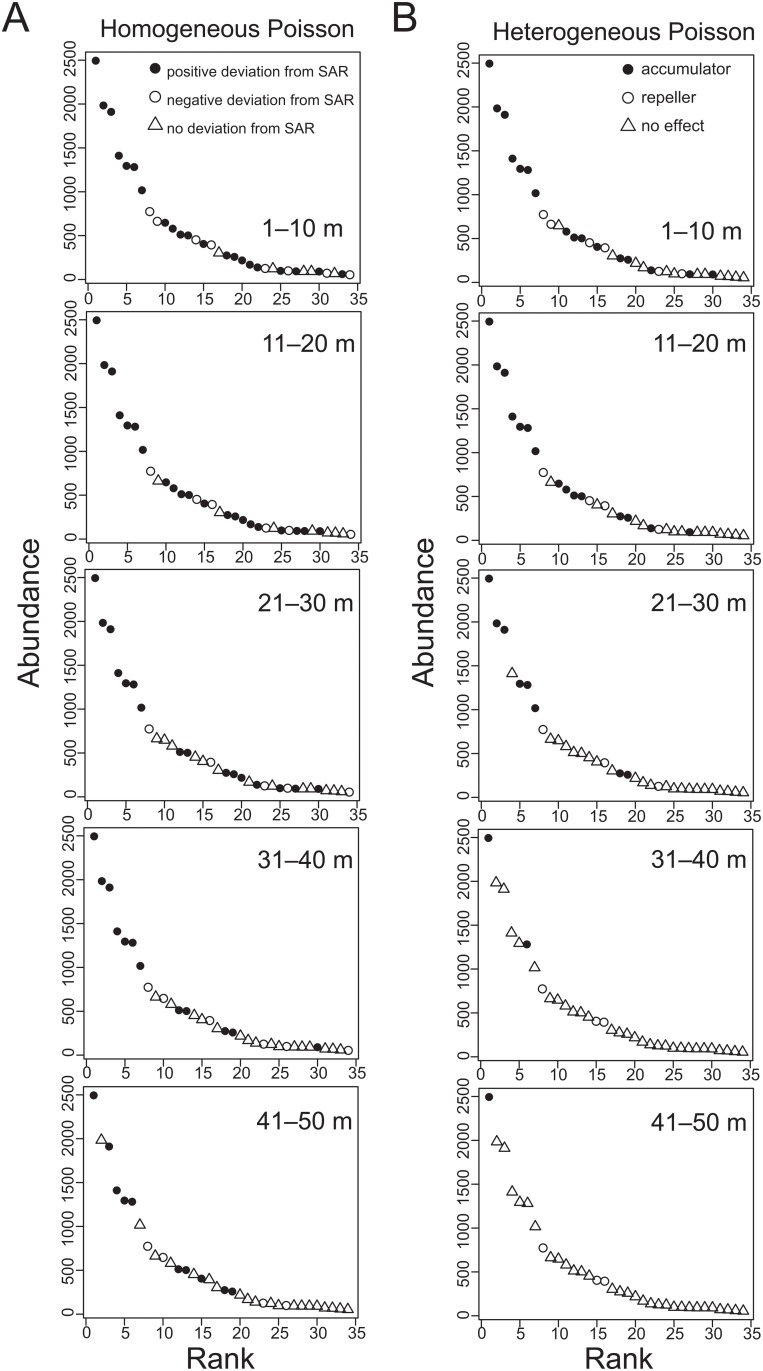
Species abundance rank distributions in relation to species types for various neighborhood distance classes. (A) Homogeneous and (B) heterogeneous Poisson null models. Solid circles, open circles, and open triangles represent positive, negative, and no deviation from conventional SAR in (A) and indicate accumulator, repeller, and no effect types in (B), respectively. Panels are arranged in order of increasing neighborhood distance from top to bottom: 1–10 m, 11–20 m, 21–30 m, 31–40 m, and 41–50 m.

We found that species with relatively high abundance ranks (i.e., abundant species) tend to show positive departures from both null models (Fig 3A and 3B). This tendency was prevalent across all spatial scales (horizontal panels in Fig 3A and 3B). For example, the 5 most abundant species (*Cyathea podophylla*, *Pyrenaria shinkoensis*, *Meliosma squamulata*, *Castanopsis cuspidata*, and *Limlia uraiana*) were classified as accumulators at almost all neighborhood distance classes (S1 Table). Importantly, these results were not statistical artifacts caused by sampling effects on ISAR estimations (S2 Fig for sensitivity analysis).

The ISAR analysis for large-small relationships produced a substantially higher proportion of accumulator species (approximately 60% of all species) than that for large-large relationships, especially at neighborhood distances <10 m (Fig 2B and 2C). That is, the dominance of accumulators in the community increases with size differences between target species and small-sized neighboring plants at neighborhood distances <10 m.

## Discussion

We show that disturbance-induced spatial heterogeneity may influence SAR patterns using an “individual-centered” approach to the species area relationship (i.e., the ISAR). This spatial heterogeneity may generally cause the dominance of species richness accumulators at small neighborhood distance scales. For example, more than 60% of species were accumulators at neighborhood scales less than 30 m. Moreover, our findings that abundant species are often richness accumulators, and that size differences increase the proportion of accumulators, suggests that plant-plant interactions (e.g., abundance- and size-dependent interactions) may act to determine the neighborhood species richness of plant communities. Our findings extend previous arguments about the distance-dependent patterns of species interactions, such as the distance-dependent Janzen-Connell hypothesis, by using ISAR approach [38–42]. In addition, our findings substantially differ from previous studies conducted in tropical forests, with our study showing that repeller species and no effect species are not prevalent across different spatial scales [13]. The prevalence of species with positive departures from the two null models in our study is probably due to recurrent disturbances in the heterogeneous monsoon rainforest [29].

### Effect of species interactions and spatial scales on ISAR

Previous studies have argued that repeller (or, at least, no effect) species are common because plant species are sessile and generally compete for space and limited resources with their neighbors [13, 43]. This phenomenon generates a negative net effect of species interactions on neighborhood species richness [13, 17, 24]. In parallel, stochasticity in survival, recruitment, and dispersal processes might lead to the independence of species and, thus, interactions that have no effect on ISAR [43]. Other factors (e.g., sprouting [26] and ectomycorrhizal networks [44, 45]) might also explain the presence of repellers and no effect species in our ISAR results. Nevertheless, the proportion of accumulators was higher than, or comparable to, those of the other types. We suggest that this phenomenon is caused by positive inter-specific interactions, intra-specific competition, and/or density- and size-dependent mechanisms operating in this frequently disturbed rainforest. For instance, a well-known distance-based density- and size-dependent hypothesis, the Janzen-Connell hypothesis, suggests that high seedling/sapling mortality occurs for individuals located near conspecific adult or large-sized plants [3]. This phenomenon is caused by species-specific negative demographic factors (e.g., pathogen infection, herbivore attack, and intense intra-specific competition; [3, 39, 40]). As such, heterospecific seedlings/saplings may establish around adult individuals at small neighborhood distances. In accordance with this hypothesis, accumulators were more dominant in large-small relationships compared to large-large relationships at small neighborhood distances (e.g., <10–30 m) in this study. This finding implies that adult plants tend to accumulate heterospecific seedlings/saplings rather than conspecific ones as their neighbors [3, 39, 40].

### Effects of species’ habitat association on ISAR

Comparison of the results of the two null models revealed the effect of species’ habitat associations on ISAR. The heterogeneous Poisson null model explains how species’ habitat preferences affect ISAR at large spatial scales (i.e., scales that are comparable to the scale of disturbance-driven canopy gaps or patches) [13, 20, 28, 46]. For instance, wind-disturbed patches, especially sites at ridges and slopes, cause spatial heterogeneity [13, 29, 30]. Data collected at our study site indicate that the wind greatly suppressed plant growth (i.e., DBH of plants was smaller than average), resulting in individual plant density and species richness being relatively high on ridges and slopes [13]. Similarly, other factors that generate spatial heterogeneity (such as flooding, landslides, light gaps, and windthrow) due to typhoons, might operate at relatively large spatial scales at our site (e.g., >50 m neighborhood distance) [29], and therefore contribute to the formation of positive deviations from SAR.

### Effects of individual abundance and size on ISAR

We hypothesized two mechanisms to explain the tendency for abundant species to be accumulators. First, density-dependent mechanisms might explain the positive relationship between population abundance and the classification of species as accumulator [3, 47]. Seedlings and saplings of dominant species, but not those of rare species, are prone to species-specific mortality factors [3, 39, 40, 47]. These density-dependent mortality factors might promote the establishment of heterospecific juveniles near the adult plants of dominant species [3, 39, 40]. Second, habitat heterogeneity (particularly spatial heterogeneity that arises from large-scale disturbances) creates spatial variation in species richness across the landscape [4, 5, 20, 22]. As such, dominant species might disproportionally occur in species-rich areas due to tolerance to disturbance and competitive superiority. These two mechanisms are not mutually exclusive, and both may explain the tendency for abundant species to be accumulators.

In accordance with the density-dependent hypothesis, ISAR patterns are size-dependent, especially for accumulators. Accumulators were more dominant in the species richness of small-sized plants in the neighborhood of large-sized focal plants (i.e., large-small relationship) than for the species richness of large-sized pants in the neighborhood of large-sized focal trees (i.e., large-large relationship). We suggest that difference exists because mechanisms associated with the distance-dependent Janzen—Connell hypothesis primarily operate between adults and seedlings/saplings [3, 39, 40]. Another possibility is that small-scale heterogeneity (i.e., micro-habitats) might affect the demographic dynamics of seedlings/saplings around adults, and might increase the proportion of accumulators locally [28, 30]. We cannot conclusively separate the effects of very small-scale heterogeneity and individual interactions on size-dependent ISAR due to the methodological assumptions and limitations of the current study. Regardless of the mechanism, our findings suggest that size differences between adjacent plants are important for determining neighborhood species richness. Our finding that abundant species and large-sized plants tend to be accumulators provides evidence for density- and size/age-dependent coexistence mechanisms underlying individual-based spatial patterns of species richness [38, 47].

## Conclusions

In this study, we used an individual-based approach to the SAR to reveal that disturbance-induced spatial heterogeneity promoted neighborhood species richness. We show that it is important to consider species interactions (such as inter-specific facilitation, intra-specific competition, especially density- and size/age-dependent effects) explicitly to improve our understanding about how spatial heterogeneity might affect conventional SAR patterns across different spatial scales. Our approach provides a novel perspective for the development of large-scale and cross-site studies, which may help resolve the contradictory results of previous studies regarding the relative importance of mechanisms with the Janzen-Connell hypothesis and disturbance in heterogeneous subtropical forests [41, 42].

## Supporting Information

S1 Fig A representative analysis of individual species-area relationship (ISAR) for *Limlia uraiana*. (DOC)

S2 Fig Sensitivity analysis of sampling (abundance) effect on identifying specific type of ISAR of the target species. (DOC)

S3 Fig Illustration of heterogeneous Poisson null model in simplified one-dimensional schematic. (DOC)

S4 Fig Diagnosis of separation of scales for individual species. (DOC)

S5 Fig The results of the heterogeneous Poisson null model for different maximum redistribution radii. (DOC)

S1 Table Categorization of all 34 species based on the individual species-area relationships under the null model of homogeneous and heterogeneous Poisson processes. (DOC)

S1 Text Sensitivity analysis of abundance effect on ISAR estimation. (DOC)

S2 Text Rationale of using heterogeneous Poisson null models to test species interactions. (DOC)
