# Supplementary material for: Individual Species-Area Relationship of Woody Plant Communities in a Heterogeneous Subtropical Monsoon Rainforest
Source: PLoS One. 2015 Apr 17;10(4):e0124539. doi: 10.1371/journal.pone.0124539 (PMC4401546; doi:10.1371/journal.pone.0124539)
Supplement: S1 Fig — (DOC) [file pone.0124539.s001.doc]

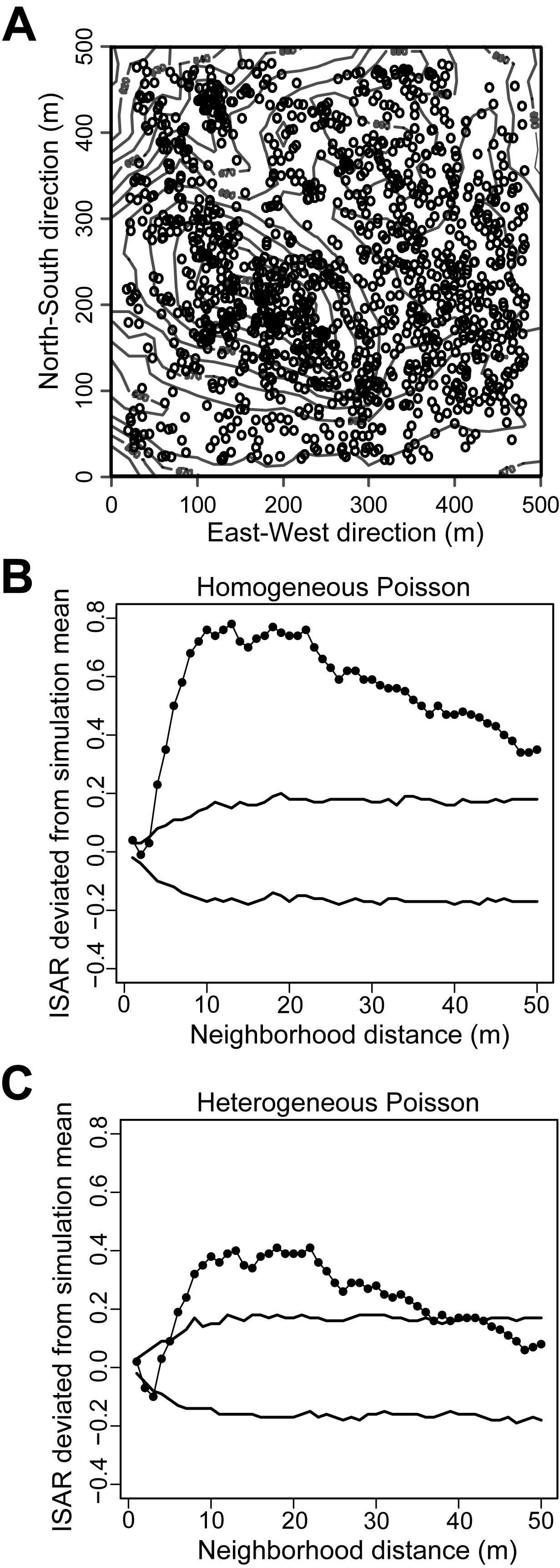


**S1 Fig. A representative analysis of individual species-area relationship (ISAR)for *Limlia uraiana*.** (A) The stem distribution map and ISAR under (B) the homogeneous and (C) heterogeneous Poisson null models. In panels (B) and (C), the solid lines with dots represent deviation between the observed ISAR and the average summary of the 199 null model simulations, and the solid lines without dots represent the simulation envelopes (i.e., the fifth lowest values of the 199 null model simulations minus the average summary).
