# Supplementary material for: Individual Species-Area Relationship of Woody Plant Communities in a Heterogeneous Subtropical Monsoon Rainforest
Source: PLoS One. 2015 Apr 17;10(4):e0124539. doi: 10.1371/journal.pone.0124539 (PMC4401546; doi:10.1371/journal.pone.0124539)
Supplement: S2 Fig — (DOC) [file pone.0124539.s002.doc]

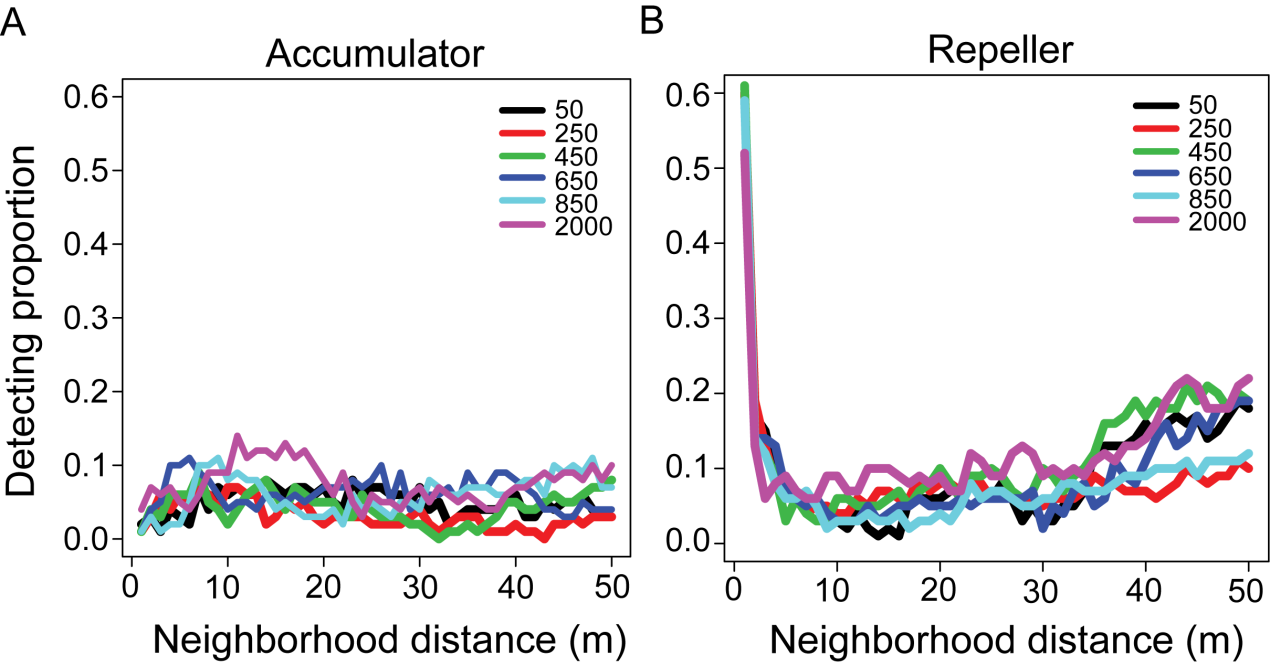


**S2** **Fig. Sensitivity analysis of sampling (abundance) effect on identifying specific type of ISAR of the target species.** Colors indicate the individual abundance levels of target species in subsampling procedure. Detecting proportion is the proportion of how many times a significant species type was detected as (A) accumulator or (B) repeller in 100 simulations for each abundance level
