# Supplementary material for: Individual Species-Area Relationship of Woody Plant Communities in a Heterogeneous Subtropical Monsoon Rainforest
Source: PLoS One. 2015 Apr 17;10(4):e0124539. doi: 10.1371/journal.pone.0124539 (PMC4401546; doi:10.1371/journal.pone.0124539)
Supplement: S3 Fig — (DOC) [file pone.0124539.s003.doc]

| 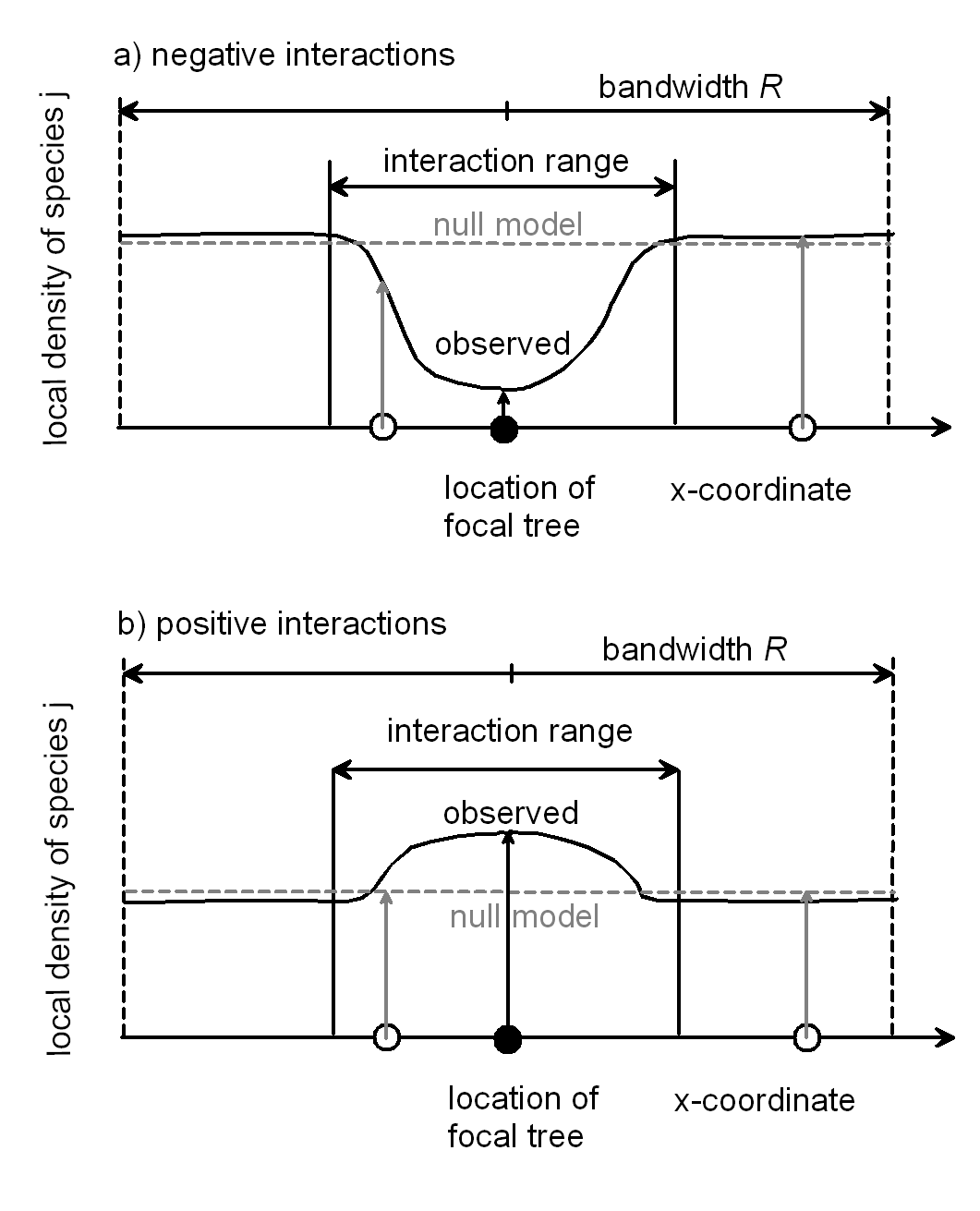 |
| --- |

**S3** **Fig. Illustration of heterogeneous Poisson null model in simplified one-dimensional schematic**. Panel (a) shows the transect in the x-direction, location of a tree of the target species (closed disk) that exerts competition to individuals of a species *j*, and local density of species *j* individuals (solid curve). Because of competition, the density of species *j* in the immediate neighborhood of the focal individual (i.e., the interaction range of competition) is lower than that of target individuals that are randomly displaced within a neighborhood *R* (open disks) from their observed location (the densities are indicated by vertical arrows). This displacement is equivalent to the heterogeneous Poisson null model where the intensity function is determined using a non-parametric estimate with bandwidth *R*. Therefore, the distribution function of the distances to the nearest *j*-neighbor observed for the target species will show negative departures from that expected under the null model. Panel (b) shows the same argument as panel (a) but for positive associations.
