# Supplementary material for: Individual Species-Area Relationship of Woody Plant Communities in a Heterogeneous Subtropical Monsoon Rainforest
Source: PLoS One. 2015 Apr 17;10(4):e0124539. doi: 10.1371/journal.pone.0124539 (PMC4401546; doi:10.1371/journal.pone.0124539)
Supplement: S4 Fig — (DOC) [file pone.0124539.s004.doc]

| 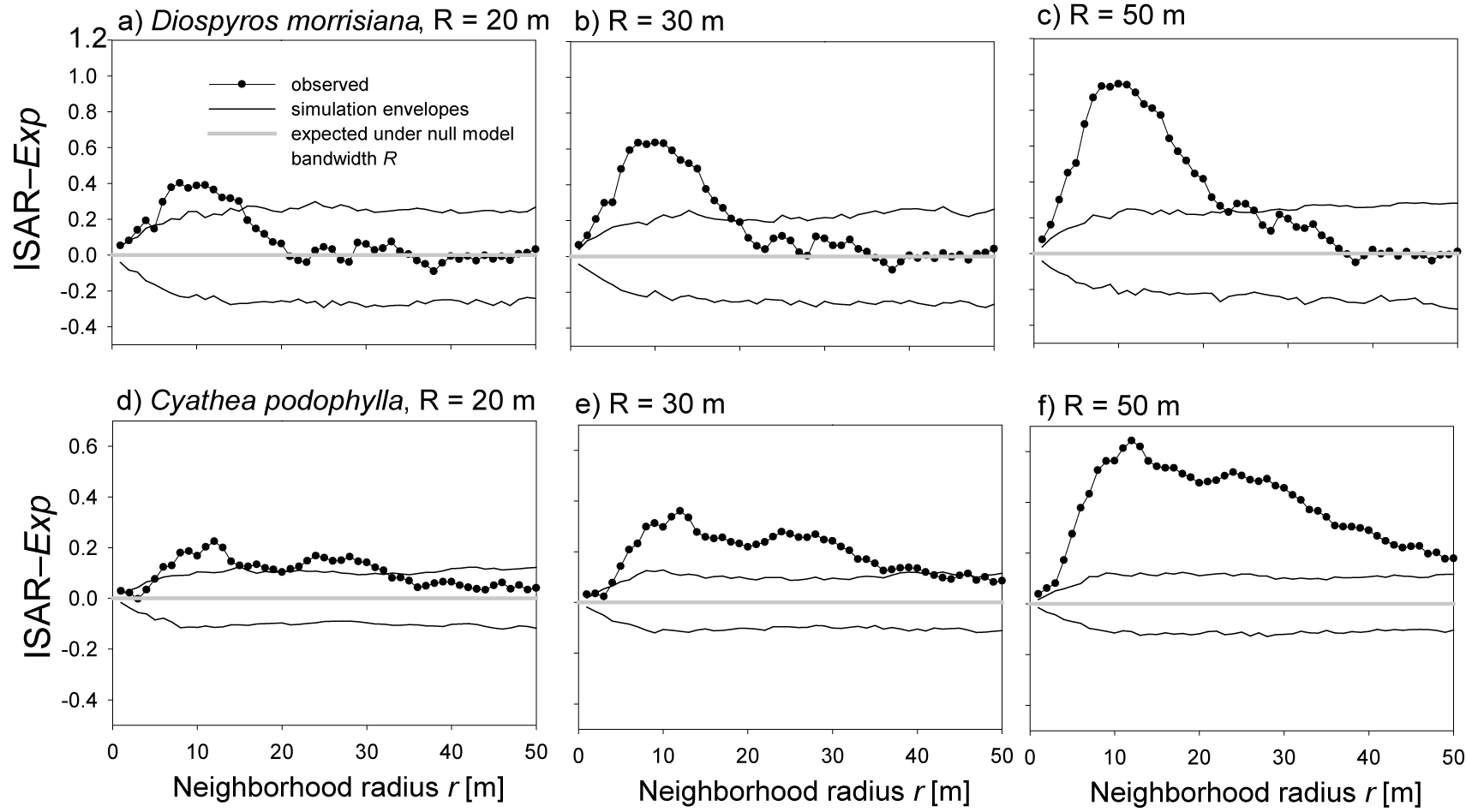 |
| --- |

**S4** **Fig. Diagnosis of separation of scales for individual species.** The species *Diospyros morrisiana* shows separation of scales where an accumulator effect occurs for neighborhoods *r* < 20 m and the ISAR agrees with the expectation at neighborhoods *r* > 20, even though the bandwidth was (a) *R* = 20 m, (b) *R* = 30 m, and (c) *R* = 50 m. The species *Cyathea podophylla*, in contrast, shows no separation of scales because the ISAR does not decline at neighborhoods *r* < *R* towards the expectation. This indicates that environmental effects determine the neighborhood diversity around individuals of the species *C. podophylla*. Note that the expectation of the null model (*Exp*) is subtracted from the observed ISAR function (closed circles) and the simulation envelopes (solid lines).
