# Supplementary material for: Individual Species-Area Relationship of Woody Plant Communities in a Heterogeneous Subtropical Monsoon Rainforest
Source: PLoS One. 2015 Apr 17;10(4):e0124539. doi: 10.1371/journal.pone.0124539 (PMC4401546; doi:10.1371/journal.pone.0124539)
Supplement: S5 Fig — (DOC) [file pone.0124539.s005.doc]

| 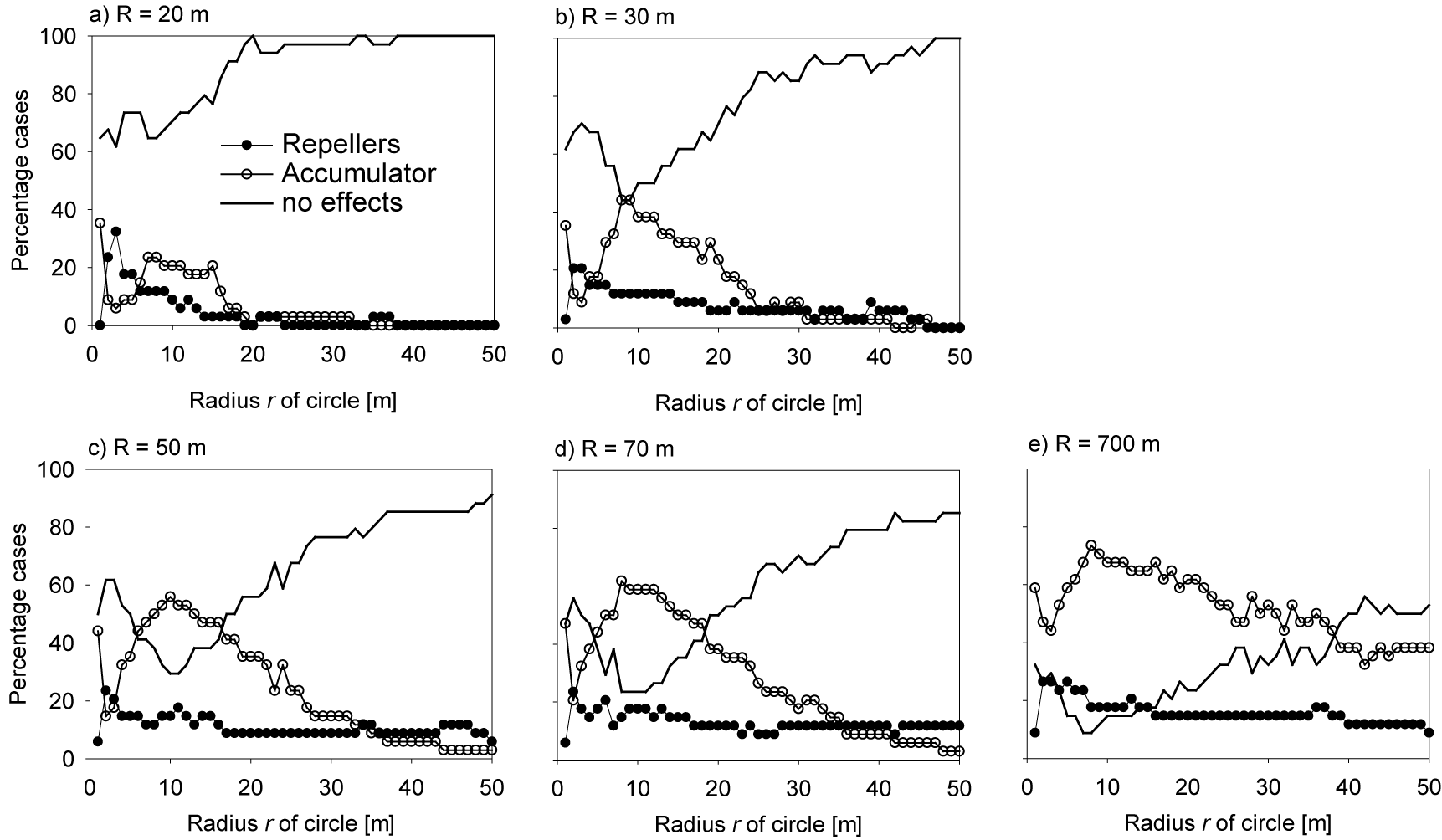 |
| --- |

**S5 Fig. The results of the heterogeneous Poisson null model for different maximum redistribution radii**. (a) 20 m, (b) 30 m, (c) 50 m, (d) 70 m, and (e) 700 m (which is almost equivalent to the homogeneous Poisson null model)
