# Supplementary material for: Individual Species-Area Relationship of Woody Plant Communities in a Heterogeneous Subtropical Monsoon Rainforest
Source: PLoS One. 2015 Apr 17;10(4):e0124539. doi: 10.1371/journal.pone.0124539 (PMC4401546; doi:10.1371/journal.pone.0124539)
Supplement: S1 Table — (DOC) [file pone.0124539.s006.doc]

**S1 Table. Categorization of all 34 species based on the individual species-area relationships under the null model of homogeneous and heterogeneous Poisson processes.** a: accumulator; r: repeller; and n: no significant effect. Abundance ranks are shown.

|  | Homogeneous Poisson | | | | | Heterogeneous Poisson | | | | |  |
| --- | --- | --- | --- | --- | --- | --- | --- | --- | --- | --- | --- |
|  | Neighborhood distance (m) | | | | | Neighborhood distance (m) | | | | |  |
| Species | 1–10 | 11–20 | 21–30 | 31–40 | 41–50 | 1–10 | 11–20 | 21–30 | 31–40 | 41–50 | Abundance rank |
| *Adinandra formosana* | A | a | a | n | n | n | n | n | n | n | 25 |
| *Castanopsis cuspidata* | A | a | a | a | a | a | a | n | n | n | 4 |
| *Cinnamomum micranthum* | A | a | a | n | n | n | n | n | n | n | 20 |
| *Cinnamomum subavenium* | A | a | a | n | n | a | a | n | n | n | 27 |
| *Cleyera japonica* | A | a | n | n | n | a | a | n | n | n | 11 |
| *Cryptocarya chinensis* | N | a | n | n | n | n | n | n | n | n | 28 |
| *Cyathea podophylla* | A | a | a | a | a | a | a | a | a | a | 1 |
| *Cyathea spinulosa* | R | r | r | r | r | r | r | r | n | n | 23 |
| *Cyclobalanopsis gilva* | N | n | n | n | n | n | n | n | n | n | 24 |
| *Cyclobalanopsis longinux* | A | a | a | a | a | a | a | a | n | n | 19 |
| *Daphniphyllum glaucescens* | A | a | a | a | n | a | n | n | n | n | 30 |
| *Diospyros morrisiana* | A | a | a | a | a | a | a | n | n | n | 12 |
| *Elaeocarpus japonicus* | A | a | n | n | a | a | n | n | r | r | 15 |
| *Engelhardtia roxburghiana* | A | a | n | r | r | n | a | n | n | n | 10 |
| *Glochidion acuminatum* | R | r | n | n | n | r | r | n | n | n | 14 |
| *Helicia formosana* | R | r | r | r | r | r | r | r | r | r | 8 |
| *Ilex formosana* | R | n | n | n | n | n | n | n | n | n | 31 |
| *Lagerstroemia subcostata* | R | r | r | r | r | r | n | n | n | n | 26 |
| *Limlia uraiana* | A | a | a | a | a | a | a | a | a | n | 6 |
| *Litsea acuminata* | A | a | a | a | n | a | a | a | n | n | 7 |
| *Machilus zuihoensis var. mushaensis* | R | n | n | n | n | r | n | n | n | n | 9 |
| *Machilus thunbergii* | A | a | a | a | a | a | a | a | n | n | 5 |
| *Machilus zuihoensis* | A | a | n | n | n | n | n | n | n | n | 21 |
| *Meliosma squamulata* | A | a | a | a | a | a | a | a | n | n | 3 |
| *Michelia compressa* | A | n | n | n | n | n | n | n | n | n | 33 |
| *Myrsine seguinii* | R | r | r | r | n | r | r | r | r | r | 16 |
| *Pasania hancei* var. *ternaticupula* | R | r | r | r | n | n | n | n | n | n | 34 |
| *Prunus phaeosticta* | A | a | a | a | a | a | a | a | n | n | 18 |
| *Pyrenaria shinkoensis* | A | a | a | a | n | a | a | a | n | n | 2 |
| *Randia cochinchinensis* | A | a | a | n | n | a | a | n | n | n | 22 |
| *Rhus succedanea* | N | n | n | n | n | n | n | n | n | n | 32 |
| *Schefflera octophylla* | A | a | a | a | a | a | a | n | n | n | 13 |
| *Symplocos theophrastifolia* | N | n | n | n | n | n | n | n | n | n | 17 |
| *Tricalysia dubia* | N | n | n | n | n | n | n | n | n | n | 29 |
