# Supplementary material for: Individual Species-Area Relationship of Woody Plant Communities in a Heterogeneous Subtropical Monsoon Rainforest
Source: PLoS One. 2015 Apr 17;10(4):e0124539. doi: 10.1371/journal.pone.0124539 (PMC4401546; doi:10.1371/journal.pone.0124539)
Supplement: S1 Text — (DOC) [file pone.0124539.s007.doc]

**S1 Text. Sensitivity analysis of abundance effect on ISAR estimation.**

*Analysis of sampling (abundance) effect on ISAR estimation*

We performed sensitivity analysis to investigate whether sampling abundances of the species would affect the detection of accumulator or repeller species. We focused on the most abundant species (2503 individuals with DBH ≥10 cm), *Cyathea podophylla*, which was detected as an accumulator. Next, we conducted a nonparametric and spatially constrained resampling process based on the spatial distribution of individuals of target species (see the next section). This process simulates the sampling (abundance) effect by reducing the total abundance of target species (*C. podophylla*) into smaller abundance levels while generally maintaining the original individual spatial structure of target species. We simulated 6 abundance levels of target species (i.e., 50, 250, 450, 650, 850, and 2000 individuals) and, for each abundance level, we repeated the simulation 100 times. In each simulation, we performed the ISAR analysis under the heterogeneous Poisson null model. This allowed us to compare the detecting power (proportions) of accumulator and repeller species over neighborhood distances at different abundance levels of the target species.

Our result shows that reducing individual abundance of target species generally did not affect the power of detecting accumulator or repeller species (S2 Fig.). In other words, the power of detecting significant interaction type did not depend on sample size. Interestingly, neighborhood distance seemed to affect the power of detecting the repeller species rather than the accumulator species. We found that it was more likely to detect the repeller species when the neighborhood distance was extremely small (<5 m) or large (>35 m) (S2 Fig.). In summary, ISAR analysis is not sensitive to the abundance of target species.

*Spatially constrained resampling method*

In order to reduce the abundance of target species while maintaining the spatial structure of individuals at the same time, we devised a nonparametric approach. First, we gridded the locations of individuals at a certain level of spatial resolution. In this study, we chose 100 m as the spatial resolution of gridding, in order to account for the broad scale variations and to compromise the assumptions in the heterogeneous Poisson null model (e.g., plant-plant interaction scale at <50 m). We found that when the gridding resolution was greater than 50 m, the results of subsampling procedures were not sensitive to the grid size. Second, we subsampled the original individuals into low abundance levels to simulate the effect of reducing sampling abundance of the target species. Third, the subsampling probability was conditioned on the spatial intensity of gridded data. That is, if individual density is higher in one spatial grid, then the subsampling probability of individuals in that grid is higher. Thus, the subsampling was conducted under a weighted probability distribution to account for the spatial variability in original distribution of target species.
