# Supplementary material for: Individual Species-Area Relationship of Woody Plant Communities in a Heterogeneous Subtropical Monsoon Rainforest
Source: PLoS One. 2015 Apr 17;10(4):e0124539. doi: 10.1371/journal.pone.0124539 (PMC4401546; doi:10.1371/journal.pone.0124539)
Supplement: S2 Text — (DOC) [file pone.0124539.s008.doc]

**S2 Text. Rationale of using heterogeneous Poisson null models to test species interactions.**

**Rationale of using heterogeneous Poisson null models to test species interactions**

Because the ISAR function contains explicit information about the spacing of the individuals of all species *j* relative to that of the target species *t*, it should reveal, similar to the *K*-function at the uni- or bivariate level, fine-scale species interactions at the community level (Wiegand et al. 2007). In the case of negative interactions between the target species *t* and a second species *j*, the density of species *j* in the immediate neighborhood of individuals of the target species (i.e., the interaction range of competition) is lower than the overall density *λ_j_* of species *j* in the entire plot (S3 Fig.). This pattern is known as repulsion (Wiegand and Moloney 2004). We can detect this pattern by randomly displacing the individuals of the target species within a local neighborhood of radius *R* that is somewhat larger than the interaction range (i.e., a heterogeneous Poisson process; Wiegand et al. 2007). Locally displaced individuals of the target species will be more frequently located at closer distances to individuals of species *j* than observed (S3 Fig.). This will be noted by the nearest neighbor distribution function *D_tj_*(*r*): it will be below the expected *D_tj_*(*r*) of this null model at small nearest neighbor distances *r*. The same argument holds true for positive association (S3 Fig.). In this case, the *D_tj_*(*r*) of the observed will be above the expected *D_tj_*(*r*) of this null model at small nearest neighbor distances *r*.

If the target species *t* exerts more negative than positive interactions to other species, the sum of all *D_tj_*(*r*) (i.e., the ISAR function) will be lower than the expectation of the heterogeneous Poisson null model. Wiegand et al. (2007) called a species exhibiting this effect a “repeller” because it reduces diversity in its immediate neighborhood. Conversely, if the target species *t* exerts more positive than negative effects on the other species, the ISAR function will be higher than the expectation. This is an “accumulator” because it increases diversity in its neighborhood (Wiegand et al. 2007). However, if the target species exerts no significant interactions, or if positive and negative interactions average out, the ISAR function will agree with the expectation of the null model.

If the environmental heterogeneity causes smooth changes in the intensity function *λ_t_*(**x**) of the target species, local relocation of individuals of the target species will follow the intensity function *λ_t_*(**x**) and therefore factor out effects of a heterogeneous environment that defines the *λ_t_*(**x**). By comparing the results of this null model to that of the homogeneous Poisson null model, we can assess the relative importance of habitat association in shaping patterns of species richness around individual plants and draw conclusions about the importance of inter-specific interactions at local scales.

Technically, we implemented the null model as heterogeneous Poisson process that uses a non-parametric Epanechikov kernel estimate of the intensity function *λ_t_*(**x**) of the target species (Wiegand and Moloney 2004; Wiegand et al. 2007; Wang et al. 2010; Wiegand et al. 2012). Because the maximal displacement distance *R* is difficult to determine *a priori*, we repeated our analyses for different values of *R* at 20, 30, 50, and 70 m (Note that the homogeneous Poisson process is the border case of the heterogeneous Poisson process with a maximal displacement radius R = 700 m). We can identify separation of scales if expected ISAR converges with expectation at some values of *r* less than *R* (S4 Fig. for 2 representative species). Thus, important information on the nature of scale-dependent effects will be revealed by a graph that shows the proportion of target species that depart from the null model as a function of distance *r*.

**Details of testing the potential impact of positive or negative species interactions**

Negative species interactions (e.g., competition) would decrease local individual density of other species. For example, if the target species *t* exerts a negative effect on species *j*, nearby *j*-individuals may have a higher risk of mortality (and/or lower probability of establishment). This further reduces the local density of *j*-individuals in the neighborhood of target individuals (S3 Fig.) and, as a consequence, leads to a lower local density of species *t* in the immediate neighborhood of *j*-individuals (say, within 10–20 m; Hubbell et al. 2001; Stoll and Newbery 2005). Thus, the nearest *j*-neighbors of target individuals are located on average at larger distances than expected by the local density of species *t* alone (measured at about 20 or 30 m neighborhood). Thus, we have “repulsion” (Wiegand and Moloney 2004). Conversely, in the case of “facilitation” (which is recognized as an important driver of succession; Connell and Slatyer 1977), the distances to the nearest *j*-neighbors tend to be smaller than expected. To detect these effects, we randomized the locations of the individuals of the target species *t*, condition on their observed local density, which is equivalent to random displacement of the focal individual within a small neighborhood *R* (S3 Fig.). If there is a negative (positive) effect of species *t* on species *j*, this will be noticed by *D_tj_*(*r*): it will be lower (higher) the expected *D_tj_*(*r*) of this null model at small nearest neighbor distances *r*.

Our null model randomly displaced the focal individuals within a small neighborhood *R* around their observed locations. We calculated the spatially varying intensity *λ*_t_(**x**) of the target species expected under this null model at any location **x** of the study area by counting all individuals that were located within the neighborhood *R* around location **x**. Clearly, the nearer the individuals to location **x**, the higher the chance that the random displacements of the null model would relocate them inside this neighborhood. In technical terms, this method to estimate *λ*_t_(**x**) corresponds to a non-parametric kernel estimate with bandwidth *R* (Stoyan and Stoyan 1994). As reported in previous studies (e.g., Wiegand et al. 2007), we used the Epanechikov kernel (Stoyan and Stoyan 1994), which estimates intensity at location **x** by counting all points within distance *R* from the local location **x** and dividing by the corresponding area. The Epanechikov kernel uses *e_R_* (*d*) that weights points located at small distances *d* from location **x** somewhat more than points that have a distance *d* slightly smaller than *R*:

$$e_{R}(d)=\left\{\begin{matrix} 2(1-\frac{d^{2}}{R^{2}}) & r\leq R \\ 0 & r>R \end{matrix}\right.$$

where *R* is the bandwidth. This produces smoother intensity estimates (Stoyan and Stoyan 1994). Note that the formula for the two-dimensional case yields

$\int_{0}^{R} e_{R}(r)2\pi rdr=\pi R^{2}$.

To simulate a realization of the heterogeneous Poisson null model (Wiegand and Moloney 2004), random coordinates within the plot are tentatively proposed, but only retained with probability λ_t_(***x***)/λ_t_* where λ_t_* is the maximal value of λ(**x**). Because the null model relocates the target individuals within distance *R* (i.e., it randomizes the actual locations at scales <*R*), it conditions on the observed local density of the target species at distances larger than *R* and will therefore detect mostly departures from the null model at distances *r* < *R* (Wiegand and Moloney 2004) (S5 Fig.). This property could detect the effects of species interactions if the bandwidth *R* is appropriately selected and separation of scales occurs (i.e., the effects of species interactions occur at finer scales than typical variation of the environment (Wang et al. 2010; Wiegand et al. 2012).

**References**

1. Connell, J. H., & Slatyer, R. O. 1977. Mechanisms of succession in natural communities and their role in community stability and organization. American Naturalist, 1119-1144.
2. Hubbell, S.P., Ahumada, J.A., Condit, R. & Foster, R.B. 2001. Local neighborhood effects on long-term survival of individual trees in a neotropical forest. Ecological Research 16, 859-875.
3. Stoll, P. & Newbery, D. 2005. Evidence of species-specific neighborhood effects in the Dipterocarpaceae of a Bornean rain forest. Ecology 86, 3048-3062.
4. Stoyan, D. & Stoyan, H. 1994. Fractals, random shapes and point fields. 1st edn. John Wiley& Sons Ltd., England.
5. Wang, X., Wiegand, T., Hao, Z., Li, B., Ye, J. & Lin, F. 2010. Species associations in an old-growth temperate forest in north-eastern China. Journal of Ecology 98, 674-686.
6. Wiegand, T. & Moloney, K.A. 2004. Rings, circles and null-models for point pattern analysis in ecology. Oikos 104, 209-229.
7. Wiegand, T., Gunatilleke, C.V.S., Gunatilleke, I. & Huth, A. 2007. How individual species structure diversity in tropical forests. Proceedings of the National Academy of Sciences of the United States of America 104, 19029-19033.
8. Wiegand, T., Huth, A., Getzin, S., Wang, X., Hao, Z., Gunatilleke, C.V.S. & Gunatilleke, I. 2012. Testing the independent species arrangement assertion made by theories of stochastic geometry of biodiversity. Proceedings of the Royal Society B: Biological Sciences 279, 3312-3320.
